# EUS-guided gallbladder drainage for distal malignant biliary obstruction: How we can evaluate clinical success

**DOI:** 10.1055/a-2644-4867

**Published:** 2025-07-24

**Authors:** Sun-Chuan Dai

**Affiliations:** 1 Gastroenterology Division, University of California San Francisco Medical Center, San Francisco, United States

**Keywords:** Endoscopic ultrasonography, Intervention EUS, Biliary tract



Innovative techniques and equipment have paved the way for interventional endoscopy to evolve in the last one to two decades beyond conventional ERCP and EUS. For patients with distal malignant biliary obstruction (DMBO) where transpapillary ERCP is unsuccessful, endoscopic ultrasound-guided choledochoduodenostomy (EUS-CDS) or EUS-guided transhepatic endoscopic retrograde cholangiopancreatography (ERCP) have provided alternatives to percutaneous drainage. When executed well and for appropriate indications, these maneuvers are equally exciting because they are beneficial to patients.

The article by Chieng M et al in the current issue of Endoscopy International Open addresses another endoscopic technique that may offer patients biliary decompression when ERCP is unsuccessful: EUS-guided gallbladder drainage (EUS-GBD). This maneuver has a proven track record for patients with acute cholecystitis who are unfit for surgical resection or require internalization of a percutaneous gallbladder drain to improve quality of life 1 . Coupling the lumen-apposing metal stent (LAMS) with an electrocautery delivery system has streamlined the procedure. Over-the-wire exchanges are minimized, procedure times are shorter, and outcomes are improved. Broadly speaking, an EUS-GBD has a shallow learning curve. There are fewer steps than a transhepatic ERCP and the target is larger compared with EUS-CDS. Naturally, this maneuver is considered in DMBO when other endoscopic options are exhausted in hopes of avoiding percutaneous transhepatic biliary drainage (PTBD).

Chieng M et al is one of many authors who report exemplary rates of technical success with EUS-GBD for DMBO, but one speculates how we should define clinical success in this scenario. In Chieng’s study, clinical success was defined as at least a 50% decrease in serum bilirubin within 3 days after EUS-GBD for malignant distal biliary obstruction, which was achieved in all 28 subjects. Despite such high rates of initial success, the authors report that not all patients normalized their bilirubin, and there were three patients who ultimately underwent percutaneous transhepatic biliary drainage (PTBD) and another had a repeat ERCP attempt at biliary stenting that was successful.

The largest study on EUS-GBD in DMBO was from Binda C et al in 2023 2 , in which 39 of 48 patients (81.3%) achieved clinical success with EUS-GBD. Clinical success in this study was defined as at least a 66.5% decrease in serum total bilirubin within 2 weeks of procedure. The authors noted that this decrease was slightly less than the means reported for EUS-CDS, possibly leading to a delay in restarting chemotherapy. There have been several systematic reviews and meta-analyses, although none comment on definitions of clinical success 3 4 .

These studies have demonstrated that EUS-GBD is technically feasible and safe. Notably, Chieng, and Binda reported a technical success rate of 100% with acceptable serious adverse event rates. However, what remains elusive is whether high clinical rates of success as defined so far translate to the big picture goal when performing EUS-GBD. Does a 50% to 66.5% reduction in bilirubin sufficiently equate to clinically significant biliary decompression for the oncologist? Do any patients with EUS-GBD have their chemotherapy regimen modified from a first-line agent or have their dose reduced because their bilirubin would not normalize? Finally, should we define clinical success by clinical course milestones instead of a percent reduction a lab value? The GALLBLADEUS study from Debourdeau A, et al 5 has broached these questions, because they reported chemotherapy reinitiation rates in their comparison between EUS-GBD and EUS-CDS. The hope is that such a trend continues in future studies.

Until we further characterize how EUS-GBD may minimize interruptions and deviations from medical therapy, prioritizing EUS-GBD as a rescue option for DMBO over PTBD (assuming PTBD can eventually be internalized) may not provide the best outcome for patients. Besides potential time lost waiting for a clinically significant bilirubin reduction that may never arrive, there are (anecdotal) instances in which partial decompression decreases duct dilation, rendering percutaneous or other endoscopic modes of biliary decompression either difficult or impossible. In addition, articles written by Chieng M, Binda C, and Debourdeau A are from seasoned experts who likely exhausted other endoscopic modes of biliary decompression before EUS-GBD. We risk misleading less experienced endoscopists who lack the support or resources to consistently execute conventional ERCP, EUS-CDS, or transhepatic ERCP into believing EUS-GBD is equally effective, potentially compromising downstream care of patients.

Interventional endoscopic ultrasound is an exciting space where adoption is widespread and rapid once new techniques demonstrate superior results. EUS-guided gastrojejunostomy, for example, has become first-line treatment for malignant gastric outlet obstruction at many centers because multiple studies suggest better outcomes compared with enteral stenting or surgery. EUS-GBD in the context of DMBO is intriguing, but because there are other established endoscopic and percutaneous options, this technique should be explored with redefined metrics of clinical success calibrated toward the clinical course of patients.
